# Supplementary material for: ComplexEye: a multi-lens array microscope for high-throughput embedded immune cell migration analysis
Source: Nat Commun. 2023 Dec 11;14:8103. doi: 10.1038/s41467-023-43765-3 (PMC10713721; doi:10.1038/s41467-023-43765-3)
Supplement: Supplementary file 1 — Supplementary information [file 41467_2023_43765_MOESM1_ESM.pdf]

ComplexEye: a multi lens array microscope for high-throughput embedded immune cell migration analysis

Zülal Cibir[1*], Jacqueline Hassel[2*], Justin Sonneck[3,4], Lennart Kowitz[3], Alexander Beer[1], Andreas Kraus[1], Gabriel Hallekamp[2], Martin Rosenkranz[2], Pascal Raffelberg[2], Sven Olfen[2], Kamil Smilowski[2], Roman Burkard[2], Iris Helfrich[5], Ali Ata Tuz[1], Vikramjeet Singh[1], Susmita Ghosh[3], Albert Sickmann[3,6,7], Anne-Kathrin Klebl[8], Jan Eike Eickhoff[8], Bert Klebl[8], Karsten Seidl[2], Jianxu Chen[3], Anton Grabmaier[2], Reinhard Viga[2#], Matthias Gunzer[1,3#]

Supplementary Information

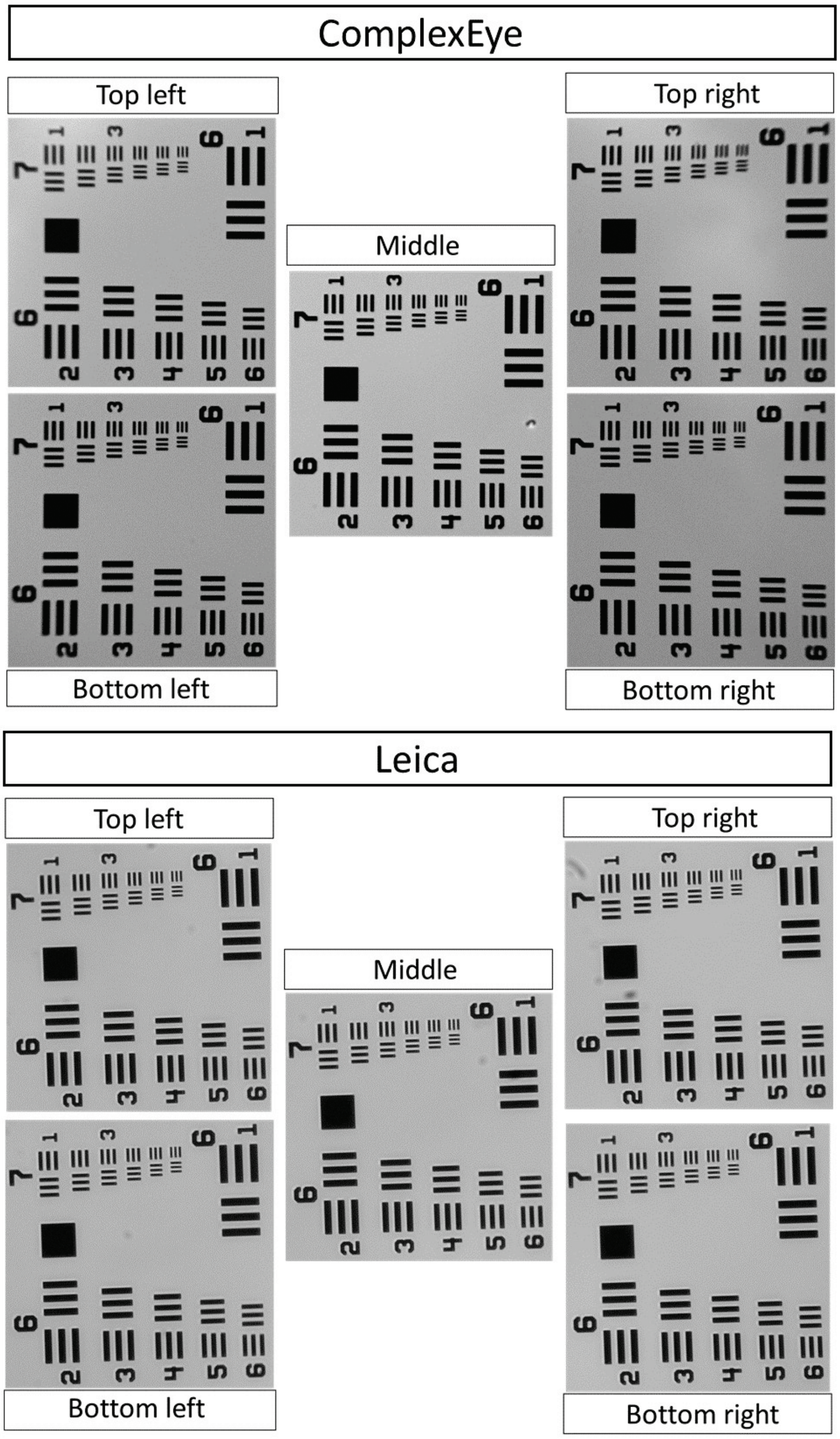

Supplementary Fig. 1 | Comparison of optical resolution. To compare the optical resolution between ComplexEye and Leica and also to quantify the contrast in different parts of the field of view of both microscopes, a positive 1951 USAF Wheel Pattern Test Target (R3L1S4P, Thorlabs) was imaged. The smallest pattern was imaged at 5 different areas of the FOV (middle, top left, top right, bottom left and bottom right). Here it was shown that both microscopes were able to resolve the smallest pattern with a resolution of 228 line pairs per millimetre (lp/mm) shown in group 7/ element 6. However, there are slight blurs in the upper parts of the top left/right fields in ComplexEye, that are absent in the Leica system pointing towards a not 100% co-aligned surface of the imaging chip with the focus area of the lens in our detection board. This experiment was not repeated.

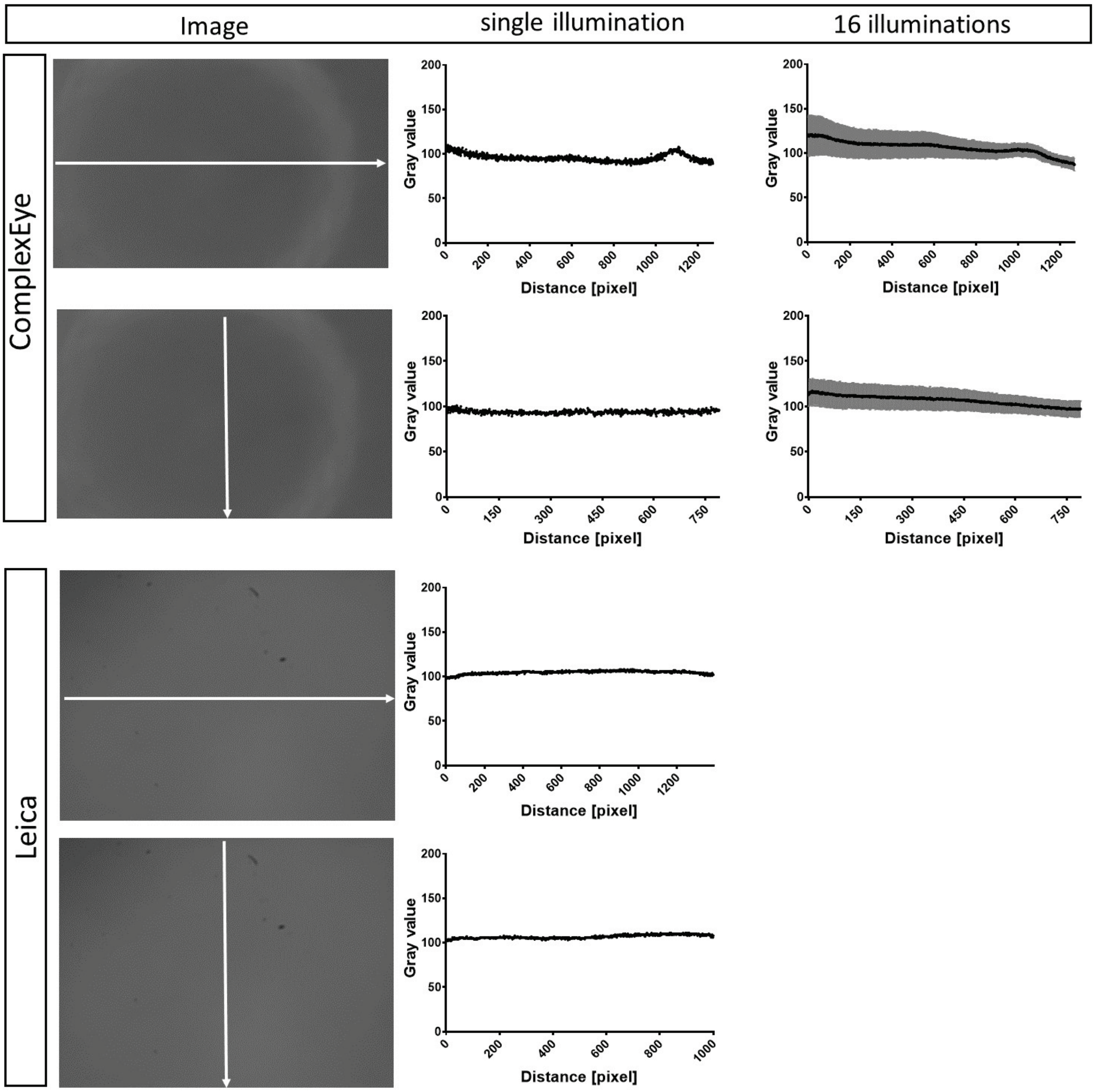

Supplementary Fig. 2 | Illumination homogeneity. To demonstrate the homogeneity of all 16 illuminations of the ComplexEye and to relate it to the conventional Leica microscope, the bottom of an empty 96-well plate was focused and imaged. The grey values of the images in horizontal and vertical lines were evaluated with Fiji and an intensity plot for the illumination was generated. For ComplexEye the intensity plot of a single illumination and of all 16 illuminations is shown in the upper part. For the vertical illumination, there was a slight gradient on the right side. Besides this, ComplexEye displayed a homogeneous illumination. The data for the 16 illuminations are shown as mean + StDev. (gray area) of all 16 illuminations. This experiment was not repeated. Source data are provided as a Source Data file.

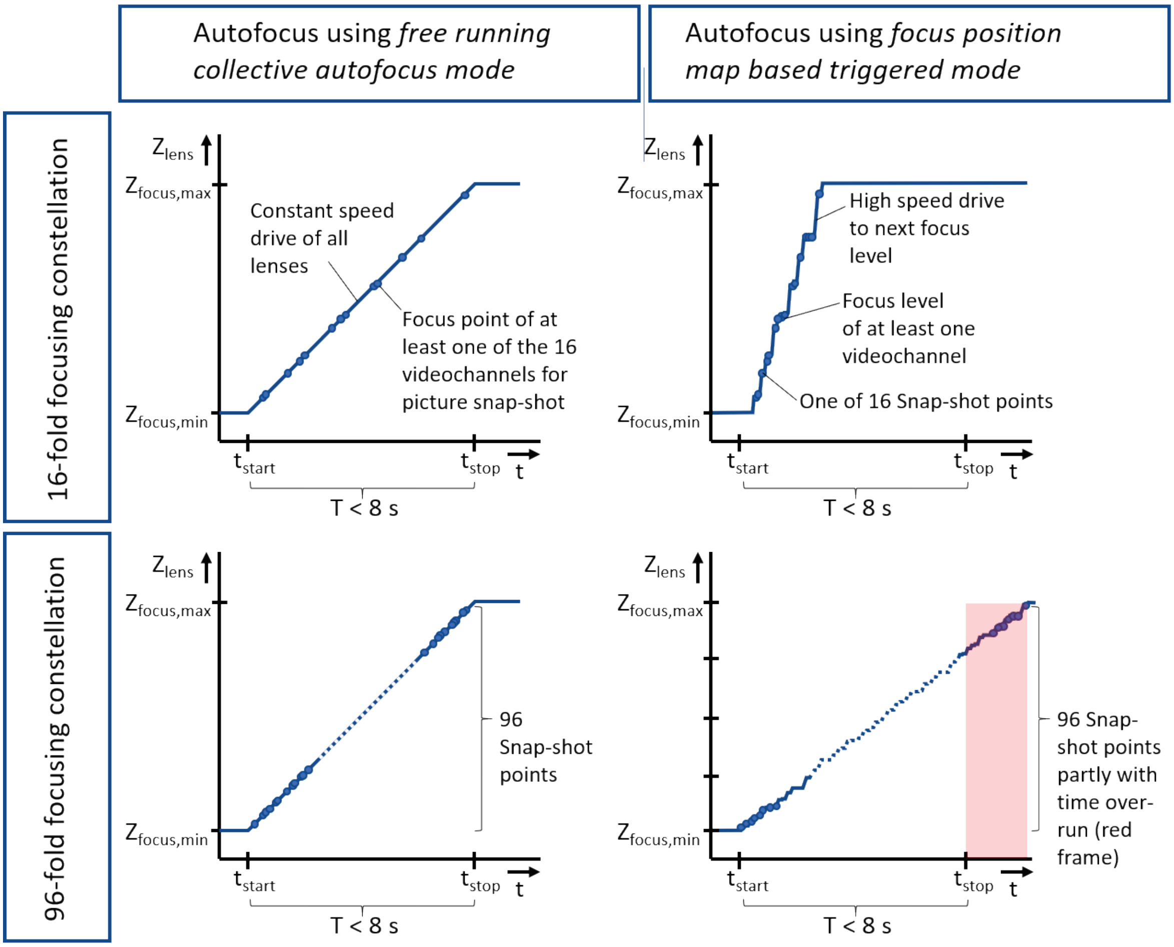

Supplementary Fig. 3 | Mode-related focusing speed considerations. Two possible autofocus variants, the "free running collective autofocus mode" and the "focus position map-based triggered mode" are shown. While in the "free running collective autofocus mode" the Z-stage is driving permanently in a constant speed and hence the total drive speed is independent of the number of recorded channels (left two diagrams), in the "focus position map-based triggered mode" (right two diagrams) each focus level requires a rest time of ~40 ms and the Z-stage pauses at each new position. Here, in conjunction with acceleration and deceleration times of the stage the "focus position map-based triggered mode" exceeds the 8 s limit in a 96 video channel constellation (lower right diagram). Hence, a 96-lens setup would only be possible with the "free-running collective autofocus mode", if staying in the 8 seconds/frame limit is required.

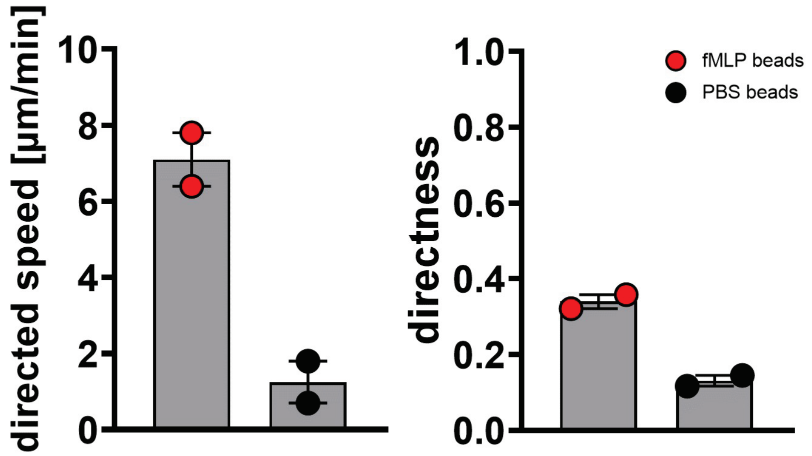

Supplementary Fig. 4 | Chemotaxis assay with beads. Freshly isolated neutrophils from healthy donors were plated on a 96-well plate and heparin beads (Adar Biotech (6024-10) incubated either with 100µM fMLP (red dots) or 1x PBS (control, black dots) for 2 hours and washed twice with 1x PBS were added to the neutrophils. Neutrophils plus beads were recorded for 1 hour with 8 seconds between frames. The tracking results clearly demonstrate that neutrophils are attracted to the fMLP beads whereas PBS beads did not recruit the cells. Bars are given as median ± interquartile range, n=2. Source data are provided as a Source Data file.

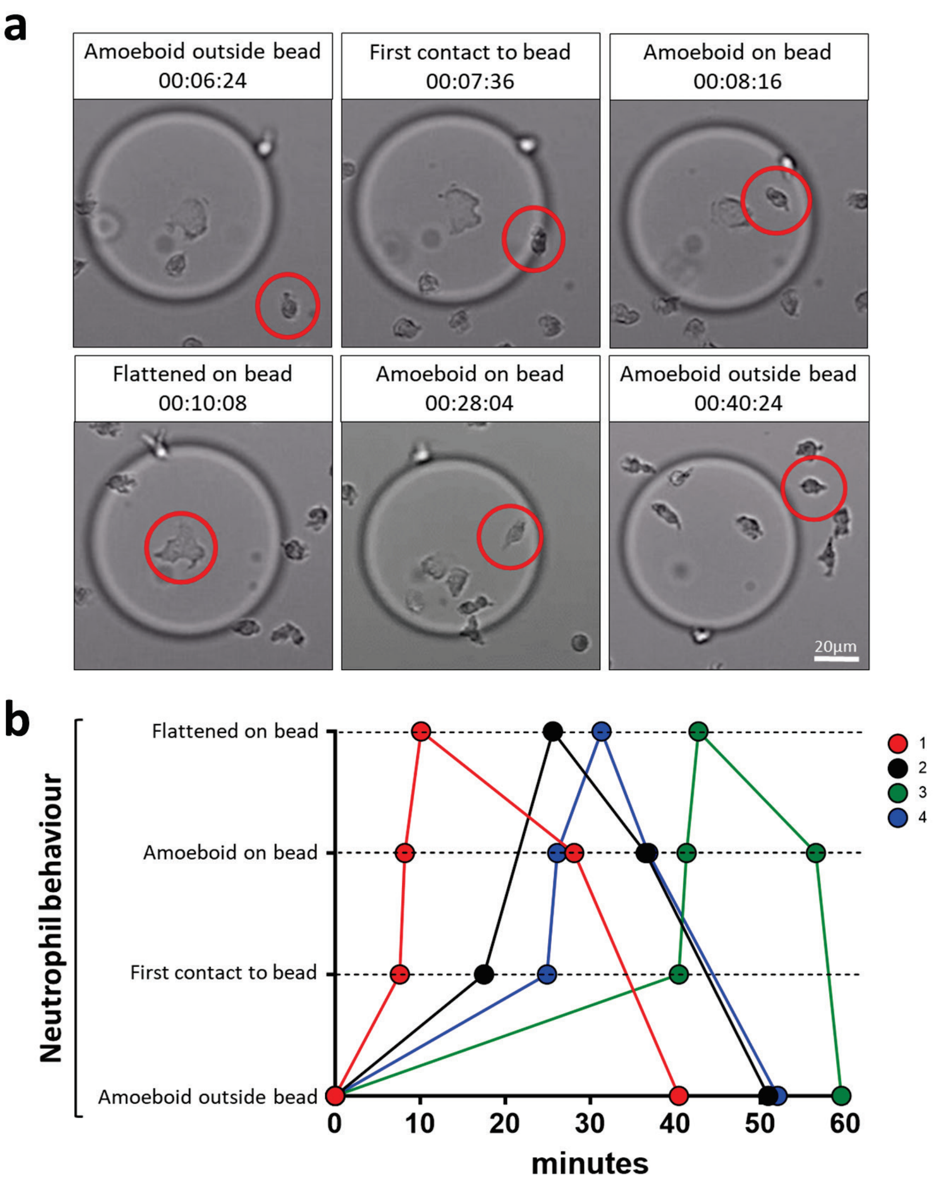

c Duration [min] of the different phases

Neutrophil	First contact	Amoeboid on bead	Flattened on bead	Ameoboid on bead
1	0.7	1.9	17.9	12.3
2	2.5	5.6	10.9	14.4
3	0.9	1.3	13.9	2.9
4	1.2	5.2	5.5	15.2

Supplementary Fig. 5 | Neutrophils change their morphology when they get in contact with fMLP beads. Freshly isolated neutrophils from a healthy donor were plated on a 96-well plate and heparin beads incubated with 100μM fMLP for 2 hours and washed twice with 1x PBS were added to the neutrophils. **a** Exemplary images of neutrophils co-cultured with fMLP beads. One individual neutrophil (red circle) was followed over time. The neutrophil displays normal amoeboid shape until it gets in contact with the bead. Upon contact, it changes the morphology and demonstrates a flattened and elongated shape. Afterwards it turns over to an amoeboid shape again and leaves the bead. Time shown in hh:mm:ss. **b** The graph displays the behaviour of 4 individual neutrophils (red, black, green and blue lines) over time. The red

line values belong to the neutrophil from the exemplary images in a. **c** The table shows the duration of each phase for the individual neutrophils in minutes. Data sets shown are based on n=1. This experiment was repeated twice and lead to the similar results.

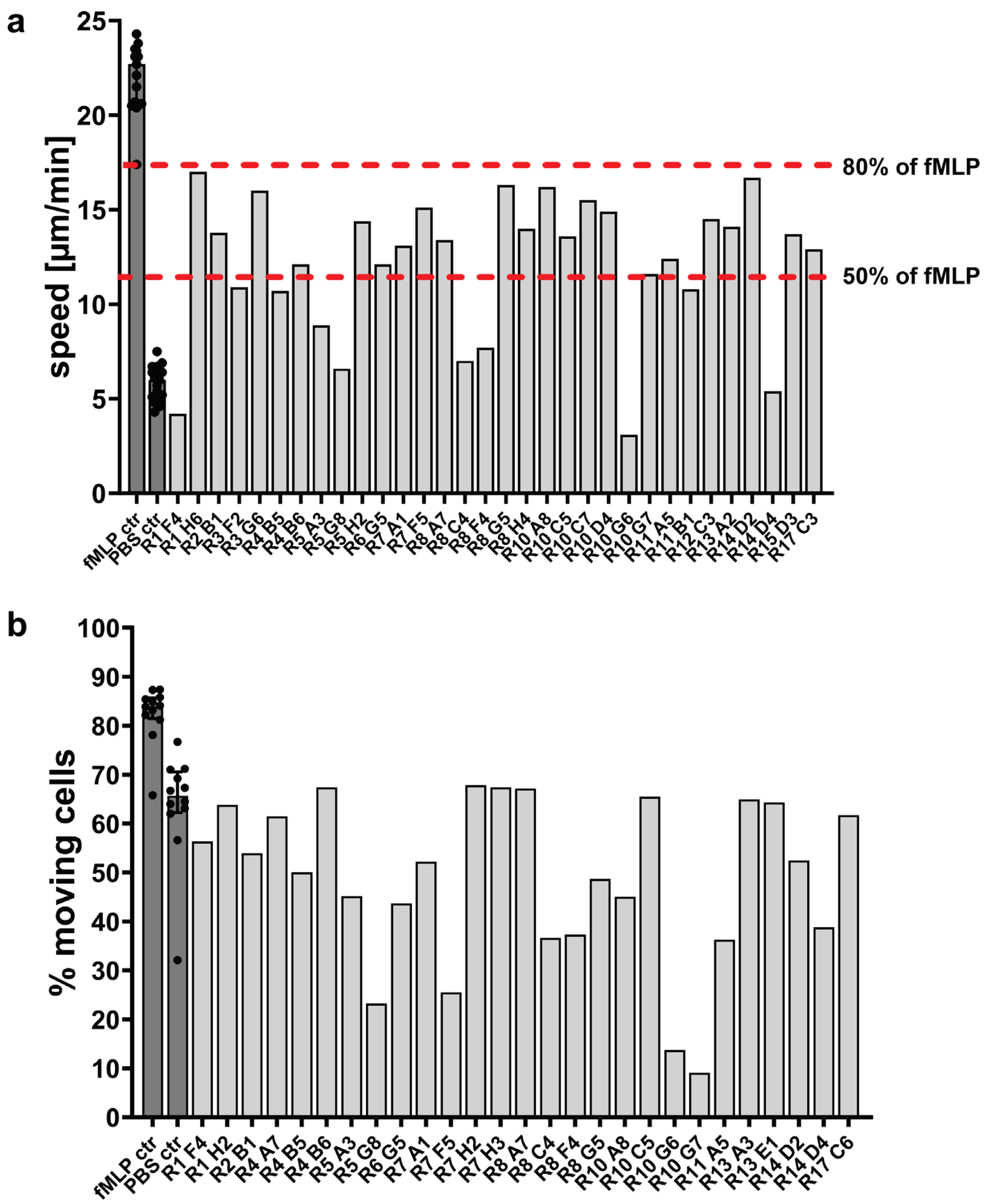

Supplementary Fig. 6 | Motility values of neutrophils modified by compounds. Freshly isolated neutrophils from a healthy donor (n=1) were plated on a 384-well plate, treated with 1 of 1,000 compounds and stimulated with fMLP. Cells were imaged for one hour with 8 seconds between frames with ComplexEye and subsequently all single cells were automatically tracked for speed in µm/min and % moving cells. Several compounds strongly decreased the fMLP-induced speed and activity of neutrophils. **a** Speed values of 32 compounds and all fMLP and PBS controls (dark gray bars). The dots in the fMLP ctr and PBS ctr bars show the speed values from all rounds in which the 32 compounds were detected. The bars indicate the mean of all cells tracked and median with interquartile range indicated by lines for fMLP and PBS. Dashed red lines indicate the 80% and 50% speed level. **b** Activity values of 27 compounds (light gray bars) and the fMLP and PBS controls. All compounds decreased the activity of neutrophils to 80% or lower. The dots in the fMLP ctr and PBS ctr bars show the activity from all rounds in which the 27 compounds were detected. The bars indicate the mean activity over the period of tracking and median with interquartile range indicated by lines for fMLP and PBS. Related to figure 5. Source data are provided as a Source Data file.

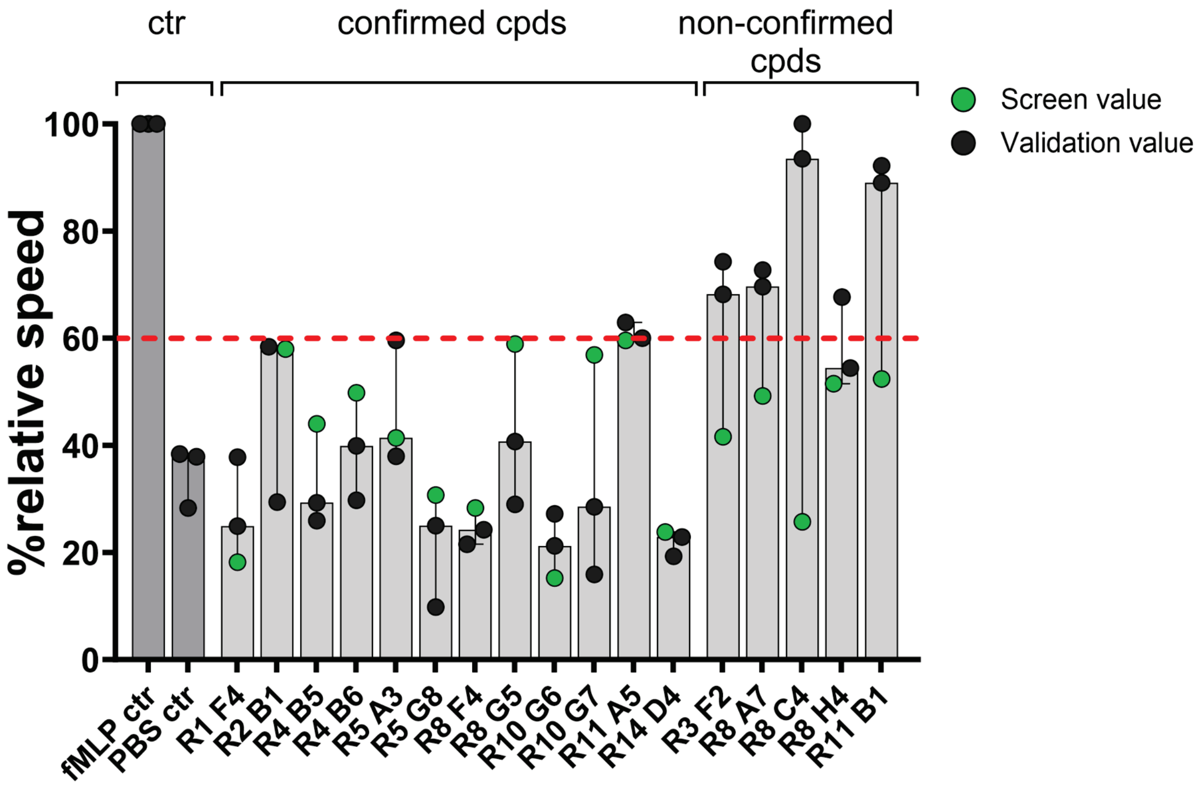

Supplementary Fig. 7 | Validation of the 17 hit compounds. Freshly isolated neutrophils from healthy donors (n=3) were plated on a 384-well plate, treated with 1 of 17 hit compounds and stimulated with fMLP. Cells were imaged for one hour with 8 seconds between frames with ComplexEye and subsequently all single cells were automatically tracked. Shown are the % relative speed data. In the first screening round (Figure 5a) all compounds were analyzed with an n=1. The values of the first screening round are shown here as green dots. Each compound was further validated with an n=2 (black dots). The fMLP and PBS controls are shown in dark gray bars. The migration inhibiting effect of 12 out of 17 compounds compared to the fMLP control could be confirmed (confirmed cpds). These 12 compounds decreased the fMLP stimulated speed of neutrophils by 40% or more compared to the fMLP control. In contrast, the migration reducing capacity of 5 out of 17 compounds could not be confirmed (non-confirmed cpds). Here, the validation values (black dots) were clearly above the 60% threshold. Data are presented as median values ± interquartile range. Source data are provided as a Source Data file.

Cpd name	Chemical target	Speed modifier	% speed reduction
R10 G6	PI3K; AKT	Yes	84.8
R1 F4	AMPK	Yes	81.8
R14 D4	Bacterial	Yes	76.2
R8 C4	RARα	Yes	74.2
R8 F4	PDI	Yes	71.7
R5 G8	CaMK	Yes	69.3
R5 A3	NF-kB	Yes	58.6
R3 F2	mTOR; PI3K	Yes	58.4
R4 B5	ROS	Yes	56.0
R8 A7	JNK	Yes	50.7
R4 B6	Phosphatase	Yes	50.2
R8 H4	JAK2; STAT3	Yes	48.5
R11 B1	EGFR	Yes	47.6
R10 G7	VCP	Yes	43.1
R2 B1	EGFR	Yes	42.0
R12 B4	EGFR	No	-
R8 G5	FLT3	Yes	40.1
R11 A5	ROCK	Yes	40.0

Supplementary Table 1. **Overview of the chemical targets of speed-reducing compounds**

Abbreviations:

PI3K/AKT: Phosphoinositide 3-kinase/Protein kinase B
AMPK: 5' AMP-activated protein kinase
RARα: retinoic acid receptor α
cAMK: Ca2+/calmodulin-dependent protein kinase
PDI: protein disulfide isomerase
ROS: reactive oxygen species
JAK2: Janus kinase 2
STAT3: Signal transducer and activator of transcription 3
mTOR: mammalian target of rapamycin
NF-κB: Nuclear factor kappa-light-chain-enhancer of activated B cells
JNK: c-Jun N-terminal kinase
EGFR: epidermal growth factor receptor
FLT3: FMS-like receptor tyrosine kinase-3
ROCK: Rho-associated coiled-coil containing protein kinase
VCP: Valosin-containing protein

R12 B4 is shown, since it targets EGFR, but unlike the other EGFR-inhibitor R2 B1 does not lead to a speed reduction but rather the opposite, a 15% speed increase over neutrophils stimulated with fMLP alone. R12 B4 and R2 B1 belong to the same substance group, while R11 B1 is of a different chemical group.

Cpd name	Chemical target	% speed increase
R6 E5	Reverse Transcriptase	26.6
R6 E3	GSK-3	24.9
R6 D4	Thyroid Hormone Receptor	23.1
R6 E7	Apoptosis	22.5
R6 D2	Apoptosis; c-Met/HGFR;	20.8
R10 A2	Apoptosis; Histone Methyltransferase	20.5
R10 E1	Apoptosis, mitochondrium	20.5
R7 F1	MMP	20.4
R7 A3	Antibiotic; Apoptosis; Autophagy	20.0

Supplementary Table 2. **Overview of the chemical targets of speed-increasing compounds**

Abbreviations:

GSK-3: Glycogen synthase kinase 3
cMet/HGFR: Tyrosine-protein kinase Met/hepatocyte growth factor receptor
MARCKS: Myristoylated alanine-rich C kinase substrate
