# Supplementary material for: ComplexEye: a multi-lens array microscope for high-throughput embedded immune cell migration analysis
Source: Nat Commun. 2023 Dec 11;14:8103. doi: 10.1038/s41467-023-43765-3 (PMC10713721; doi:10.1038/s41467-023-43765-3)
Supplement: Supplementary file 3 — Description of Additional Supplementary Files [file 41467_2023_43765_MOESM3_ESM.pdf]

DESCRIPTION OF ADDITIONAL SUPPLEMENTARY FILES

Supplementary Movie 1. ComplexEye 3D overall view

3D animation of the central components of the ComplexEye. Related to figure 2b.

Supplementary Movie 2. ComplexEye 3D view of the microscope unit

3D animation with a detailed view of the imaging units and the 8 mm lenses. Related to figure 2b.

Supplementary Movie 3. Long-term recording of migrating cancer cells

CT26 colorectal carcinoma cells were incubated on 96-well flat bottom plates and imaged with ComplexEye at one frame per minute for 11 hours.

Supplementary Movie 4. fMLP treated neutrophil migration

Freshly isolated neutrophils from healthy donors were plated on a 96-well flat bottom plate and recorded with the ComplexEye. Shown is a one-hour movie of neutrophils stimulated with fMLP. Real time between frames is 8 seconds.

Supplementary Movie 5. ComplexEye comparison with a conventional microscope

Freshly isolated neutrophils from healthy donors were plated on two 96-well flat bottom plates and recorded simultaneously at the ComplexEye and a commercial microscope from Leica. Neutrophils were treated with PBS, fMLP, CXCL1 and CXCL8 and were imaged for one hour with 8 seconds between frames. Note the comparable optical performance and migratory behavior of neutrophils. Related to figure 3a.

Supplementary Movie 6. Chemotaxis assay with beads

Freshly isolated neutrophils from healthy donors were plated on a 96-well plate and heparin beads incubated either with 100µM fMLP or 1x PBS (control) for 2 hours and washed twice wi th 1x PBS were added to the neutrophils. Neutrophils plus beads were recorded for 1 hour wit h 8 seconds between frames. Shown are neutrophils co-cultivated with fMLP and PBS beads. Neutrophils are recruited to the fMLP bead, whereas the PBS bead is ignored. Related to sup plemental figure 4/5.

Supplementary Movie 7. Compounds gradually decreasing fMLP-induced speed

Freshly isolated neutrophils from healthy donors were plated on a 384-well flat bottom plate, treated with 1 of 1,000 compounds and stimulated with fMLP. Several compounds strongly decreased the fMLP-induced speed of neutrophils. Shown are four compounds gradually decreasing the speed from 80-20%. As a comparison the fMLP and PBS control movies are shown. Related to figure 4b-c. Compound names correspond to the positions on the migration screen shown in figure 4b.

Supplementary Movie 8. Compounds gradually decreasing fMLP-induced activity

Freshly isolated neutrophils from healthy donors were plated on a 384-well flat bottom plate, treated with 1 of 1,000 compounds and stimulated with fMLP. Several compounds strongly decreased the fMLP-induced activity of neutrophils. Shown are four compounds gradually decreasing the activity from 80-20%. As a comparison the fMLP and PBS control movies are shown. Related to figure 4d. Compound names correspond to the positions on the migration screen shown in figure 4b.

Supplementary Movie 9. Compounds modifying neutrophil shape

Freshly isolated neutrophils from healthy donors were plated on a 384-well flat bottom plate, treated with 1 of 1,000 compounds and stimulated with fMLP. Several compounds strongly modified the shape of neutrophils. Shown are three compounds differently changing neutrophil morphology. As a comparison the fMLP movie with typical migratory cell shape of neutrophils is shown. Related to figure 5d. Compound names correspond to the positions on the migration screen shown in figure 4b.

Supplementary Data 1. Overview of the tracking values for each compound and the respective controls.

This Excel sheet shows all evaluated tracking values for the tested compounds and the respective controls (PBS ctr, DMSO ctr, DMSO + fMLP ctr and fMLP ctr, shown in blue colors). Included are the number of total tracks, the number of valid tracks, mean speed in µm/min, % relative speed, activity in % and % relative activity. Thereby, valid tracks are determined by two parameters: minimum track duration and movement threshold. For neutrophils, the minimum track duration is defined as 1 min (8 frames or more) and the movement threshold 8 µm (one cell diameter or more). All movies were recorded for 1 hour with 8 seconds between frames and thus consisted of 450 frames.
